# The Gut–Brain Axis and Its Role in Controlling Eating Behavior in Intestinal Inflammation

**DOI:** 10.3390/nu13030981

**Published:** 2021-03-18

**Authors:** Gordon William Moran, Gita Thapaliya

**Affiliations:** 1 National Institute of Health Research Nottingham Biomedical Research Centre, University of Nottingham, and Nottingham University Hospitals NHS Trust, Nottingham NG7 2UH, UK; 2 Division of Child & Adolescent Psychiatry, Department of Psychiatry and Behavioral Sciences, Johns Hopkins University School of Medicine, Baltimore, MD 21287, USA; gthapal2@jhmi.edu

**Keywords:** enteroendocrine cells, enteroendocrine peptides, gut hormones, gut–brain axis, intestinal inflammation, eating behavior

## Abstract

Malnutrition represents a major problem in the clinical management of the inflammatory bowel disease (IBD). Presently, our understanding of the cross-link between eating behavior and intestinal inflammation is still in its infancy. Crohn’s disease patients with active disease exhibit strong hedonic desires for food and emotional eating patterns possibly to ameliorate feelings of low mood, anxiety, and depression. Impulsivity traits seen in IBD patients may predispose them to palatable food intake as an immediate reward rather than concerns for future health. The upregulation of enteroendocrine cells (EEC) peptide response to food intake has been described in ileal inflammation, which may lead to alterations in gut–brain signaling with implications for appetite and eating behavior. In summary, a complex interplay of gut peptides, psychological, cognitive factors, disease-related symptoms, and inflammatory burden may ultimately govern eating behavior in intestinal inflammation.

## 1. Introduction

Malnutrition represents a major problem in the clinical management of the inflammatory bowel diseases (IBD), Crohn’s disease (CD), and ulcerative colitis (UC). Malnutrition may be attributed to poor nutritional intake, micronutrient deficiencies due to impaired utilization or loss of nutrients, malabsorption due to mucosal inflammation or resection [1]. Further, the negative nitrogen balance observed in CD is related to increased protein catabolism rates [2] in response to increased protein requirements attributed to intestinal and systemic inflammation (e.g., acute-phase protein, pro-inflammatory cytokine, and fecal calprotectin FCP production). Several other factors such as disease burden, appetite loss, disordered eating, and other associated symptoms such as nausea, diarrhea contribute to malnutrition with a negative impact on IBD patients’ quality of life. In this review, we will present the current understanding of the effect of intestinal inflammation on gut–brain signaling and eating behavior.

## 2. Methods

A literature search was performed on PubMed (National Library of Medicine) using search terms: intestinal inflammation, inflammatory bowel disease, Crohn’s disease, Ulcerative colitis, enteroendocrine cells, enteroendocrine cell peptides, gut hormones, brain MRI, gut–brain axis, and eating behavior. All research articles were considered based on the priority scale as follows: systematic reviews, and meta-analyses, randomized controlled trials (RCTs), human observational studies, case studies, animal and in vitro studies. We selected publications between January 1978 and December 2020. Of the selected articles, the full texts and the references were reviewed, and if the reference list contained eligible articles those were also included. All publication not written in the English language were excluded.

## 3. Eating Behavior in IBD

Malabsorption and catabolism are considered to be the major contributors to weight loss in IBD. However, suppressed appetite, early satiety, and reduced food intake are also clinically relevant in the etiology of nutritional abnormalities in IBD patients [3]. Appetite can be described as “the internal driving force for the search, choice, and ingestion of food” [4]. The intake of food results in satiation that decreases the drive to eat thereby terminating an eating episode, which leads to a feeling of satiety that inhibits hunger and determines the quantity of food consumed at the next eating episode [5]. Food intake is governed by complex neurohormonal appetite networks involving homeostatic and hedonic factors. Disordered eating might be linked with a disruption in homeostatic and hedonic balance. Presently, our understanding of the cross-link between eating behavior and IBD is still in its infancy. Patients with gastrointestinal disorders are at a higher risk of disordered eating patterns such as meal skipping, food restriction, and binge eating [6,7]. A survey investigating dietary beliefs and behaviors in IBD patients showed that 48% believed diet was the initiating factor in IBD and 57% believed it could trigger a flare [8]. Certain foods such as (sugary food, coffee/tea, carbonated beverages, milk/milk products, raw vegetables/fruits, alcohol, fatty food, and spicy food) were implicated in worsening symptoms, hence avoided to reduce symptoms [8,9,10].

A disordered eating behavior may be described with a two-path theoretical model [6,7]. The first pathway describes feelings of heightened anxiety about unfamiliar foods and overestimation of the negative consequences linked with their disease. The second pathway describes the type of behavior that employs techniques to lose weight to counteract weight gain induced by medical or dietary interventions. Disordered eating in CD patients during active disease has been previously reported [11]. CD patients reported greater anxiety/depression, greater emotional eating, lower positive mood, greater binge eating tendencies, lower craving control, and greater craving for sweet and savory foods. Reduced protein consumption was found in males and carbohydrates in females [11]. However, other studies have shown no difference in energy intake but suboptimal micronutrient intakes in both active and inactive CD [12,13]. Anxiety about food intake in CD patients may lead to restriction of food groups to minimize fear of worsening symptoms [7]. Anxiety and depression in IBD are complex issues that could be a manifestation of several factors, e.g., lower socioeconomic status [14], poor quality of life due to chronic fatigue [15], pain [16], and extraintestinal manifestations [17]. CD patients may exhibit increased food monitoring behaviors, often seen in people with gastrointestinal disorders [6,7,11]. The role of energy-dense food in alleviating low mood also called “comfort eating” has been demonstrated in healthy individuals, where neural responses in key hedonic brain regions, including midbrain, dorsal and ventral striatum, left anterior cingulate cortex (ACC) right hippocampus, and homeostatic regions such as the hypothalamus and brainstem were attenuated by a fatty acid infusion in an experimentally induced sad mental state [18]. CD patients may have stronger hedonic desires for food and emotional eating patterns possibly to alleviate symptoms of low mood, anxiety, and depression [11]. A recent study found an association between the inflammatory potential of diet, as assessed by Dietary Inflammatory Index (DII) [19] and disease severity in CD but not in UC [20]. Palatable but, pro-inflammatory foods found in the Western diet may provide immediate reward but worsen CD severity in the long term. Longitudinal studies are needed to investigate the effect of diet on disease course.

Personality traits such as impulsivity may also influence eating behaviors in IBD patients. Impulsivity has been described as 3 distinct psychological manifestations, i.e., impulsive choice, impulsive action, and reflection impulsivity [21]. An impulsive choice represents a measure of reward sensitivity whereby a person tends to accept small or immediate rewards instead of larger future outcomes [22]. Impulsive action represents the inability to inhibit an inappropriate reaction to prepotent stimuli [22]. Reflection impulsivity describes the failure to gather essential information and evaluate this information before making a decision [23]. A study that examined different personality traits in IBD [24], found that impulsive choice as assessed by the Eysenck Impulsivity Inventor [25], was higher in IBD patients (both with UC and CD) compared with controls. Further, elevated impulsivity was associated with low levels of physical and mental health and poorer quality of life in IBD patients, which was more characteristic of the female gender [24]. Another study [26], which investigated “impulsive sensation seeking” (ImpSS) personality characteristic in IBD found that smokers with CD had higher ImpSS scores than smokers with UC. Smokers with CD also scored higher on the nicotine dependence scale than smokers with UC [26]. Impulsivity is a personality trait that predisposes toward rewarding behavior, which may explain smoking behaviors in CD due to the rewarding properties of nicotine that is linked with the mesolimbic dopaminergic system [27]. The mesolimbic dopaminergic system also dictates the reward and pleasure-driven liking and wanting of food [28]. If inhibitory control is impaired, the preference for palatable foods becomes more appealing as an immediate reward than future health concerns. Taken together, a complex phenomenon involving reward and impulse-driven food choices to ameliorate symptoms such as low mood, abdominal pain, and chronic fatigue may influence eating behaviors in IBD.

## 4. Physiology of Appetite Regulation

The enteroendocrine gut–brain axis plays a critical role in the homeostatic regulation of appetite. The enteroendocrine system is the largest endocrine organ in the human body and it is made up of enteroendocrine cells (EEC) [29]. EEC are found distributed as single cells throughout the intestinal tract and are located within intestinal crypts and villi and represent 1% of cells lining the intestinal epithelium [29]. Gastrin, ghrelin, somatostatin, cholecystokinin (CCK), glucose-dependent insulinotropic peptide (GIP), glucagon-like peptide (GLP-1), and peptide YY (PYY) are some of the peptides secreted by EEC [30]. Ghrelin is secreted by the stomach and enhances appetite [31], whereas CCK, PYY, and GLP-1 suppress appetite [32]. EEC act as sensors of the luminal nutrient content either in a classical endocrine fashion or by paracrine effect on proximal cells, notably vagal afferent fibers [33], and secrete peptides and amines that regulate postprandial gut secretion and motility [34]. Food-induced stretching of the stomach muscle wall signals through the gastric mechanoreceptors and activates the vagus nerve. The vagus nerve is made up of both afferent fibers (visceral sensory nerve) and efferent fibers (motor nerves) [35]. Peripheral vagal afferent fibers are scattered along with the mucosal and submucosal layers of the digestive tract. The EEC peptides act on vagal afferents in the gastrointestinal tract directly relaying signals to the CNS through the *nucleus tractus solitarius* (NTS) [36], to regulate ingestive behavior [37]. Some of the key EEC peptides (CCK, PYY, GLP-1, and ghrelin) are described below.

### 4.1. CCK

CCK is one of the first endogenous gut hormones documented to affect appetite and a neuropeptide that is abundantly found in the CNS [38]. CCK is synthesized and released from the I cells of the proximal duodenum and jejunum in response to luminal content, particularly, protein and fat [39]. It has a short half-life of 1–2 min (i.e., rapidly deactivated after release). Long-chain fatty acids of chain length C12 can stimulate I cells to release CCK [40] via G-protein-coupled receptor 40 [41]. CCK exerts its effects via receptors CCK-1 and CCK-2. CCK-1 is found in the gastrointestinal tract and CCK-2 is expressed in the CNS [5]. CCK binds to its CCK-1 receptor on the sensory terminals of the vagus nerve relaying satiety signals to the arcuate nucleus (ARC) in the hypothalamus [42]. Peripheral administration of CCK reduces appetite and meal size in rodents and humans [43]. CCK-1 receptor knockout rats exhibit an increase in food intake and develop an obese phenotype, attributed to the overexpression of neuropeptide Y (NPY) [44]. A high dose of intravenous administration of CCK can induce nausea and abdominal discomfort, contributing to the reduction in food intake [45]. CCK levels rise over 10–30 min after food ingestion and act to stimulate gall bladder contraction, pancreatic secretions, and delayed gastric emptying, collectively contributing to the feeling of fullness and satiety [39]. Central administration of CCK agonists via intracerebroventricular injections reduces food intake in rodents [46]. Finally, the central administration of CCK with leptin induces weight loss in rodents indicating the role of CCK in enhancing the effect of leptin in the regulation of appetite [47].

### 4.2. GLP-1

GLP-1 is released from the L cells in the distal small intestine and proximal colon proportionately to ingested carbohydrate load [48]. GLP-1 is released rapidly into the circulation with a biphasic response to food intake mainly carbohydrates (CHO). It is released 10–15 min postprandially and again peaks after 30–60 min [5]. The initial release of GLP-1 is vagally mediated [49] or due to stimulation of GLP-1/GIP co-expressing K cells. The later release of GLP-1 is due to direct stimulation of ileal L cells [50]. Peripheral and central administration of GLP-1 and GLP-1 receptor agonists (exendin-4) show reduced food intake in rodents [51]. The GLP-1 receptors in the CNS rather than the vagus nerve contribute to the complete anorectic response to the GLP-1 agonist liraglutide, suggesting that GLP-1 receptors in the CNS are involved in weight loss [52]. GLP-1 signals converge on the NTS and in the area postrema (AP) and directly activate pro-opiomelanocortin/cocaine- and amphetamine-regulated transcript (POMC/CART) and indirectly inhibit the NPY/agouti-related peptide (NPY/AgRP) via GABAergic transmission to reduce food intake [53]. Further, fluorescently labeled liraglutide penetrates the brain activating GLP-1 receptor-expressing neurons in the ARC to induce weight loss in rodents [54]. Liraglutide alters brain activity in response to highly palatable food cues in Type 2 diabetic patients (T2DM) [55]. This finding is in agreement with another study that showed that liraglutide improved hypo-responsiveness to palatable food in obese T2DM patients relative to lean healthy controls, indicating the role of GLP-1 in the hedonic control of food intake [56]. GLP-1 is also an incretin hormone that stimulates insulin biosynthesis by acting on pancreatic β cell receptors in response to glucose load and regulates glucose homeostasis [43]. GLP-1 exerts its satiating effects via ileal brake, a feedback mechanism where distal intestinal signals inhibit proximal gastrointestinal motility and gastric emptying [39]. Finally, the presence of GLP-1 receptors in the (AP) has been linked to side effects, i.e., nausea and vomiting, of GLP-1 analogues [57].

### 4.3. PYY

PYY is synthesized and released from the L cells in the ileum and colon in response to food intake, especially fat and fermentable CHO [38]. PYY concentrations are low in a fasting state and rise postprandially, in proportion to the energy intake signaling the appetite-regulating circuits to subsequently reduce food intake [58]. As with GLP-1, PYY also shows a biphasic response with levels rising within 30 min after meal intake [59]. The early increase in PYY is CCK mediated [60] while the later increase is due to direct nutrient stimulation of L cells in the ileum [61]. PYY binds to G protein receptors Y1 and Y2 [5] and exerts its anorectic effects by acting on the Y2 receptor (Y2R), as evidenced by the inhibition of food intake in response to Y2R agonist [62] with this effect being dampened by Y2R antagonist [63]. Y2R is expressed on both NPY and (AGRP) orexigenic neurons in the hypothalamus and induces its anorectic effects by inhibiting NPY/ARGP neurons via Y2R and activating the anorexigenic POMC neurons [64]. Further, Y2R is also expressed by the vagal afferent terminals; the peripheral administration of PYY induces anorectic effects via the arcuate neuronal activation, and vagotomy or transection of hindbrain-hypothalamic pathways abolishes this effect [65]. In addition to PYY’s direct central effect on appetite, PYY also modulates gut motility via ileal brake, through delay in gastric emptying and gastrointestinal transit time [66], resulting in a sensation of fullness and satiety [58]. Supraphysiological intravenous infusions of PYY have shown to induce nausea in humans, thereby having a negative impact on food intake [67].

### 4.4. Ghrelin

Ghrelin is an orexigenic hormone secreted by X/A cells in the mucosa throughout the length of the GI tract, with the highest abundance in the gastric fundus in the stomach. Circulating ghrelin is significantly increased during fasting and attenuated following a meal. Central and peripheral administration of ghrelin stimulates food intake leading to an increase in bodyweight [68]. These effects are diminished after vagotomy, indicating a gut–brain signaling pathway [31]. The appetite stimulatory action of ghrelin is mediated through direct stimulation of the orexigenic AgRP/NPY neurons and concomitant inhibition of the anorectic POMC/CART neurons in the ARC [69]. In addition to its orexigenic effects, ghrelin also acts as an anabolic hormone that drives lipogenesis [70]. Ghrelin is also a key regulator of glucose homeostasis, by protecting against hypoglycemia via growth hormone (GH) release from the anterior pituitary [71], increasing glucagon secretion [72], and blocking insulin secretion [73]. Ghrelin is a critical hormone implicated in stress response. Ghrelin may target the *ventral tegmental area* (VTA), hippocampus, and amygdala to modulate reward processes by enhancing feeding during a stress response, as a coping mechanism to protect against damage associated with chronic stress [74]. Ghrelin also acts as an anti-inflammatory hormone, inhibiting acute phase protein and pro-inflammatory cytokine expression [75]. The upregulation of ghrelin observed in acute and chronic inflammatory conditions has been attributed to a host’s attempt to restore homeostatic balance via regulating food intake, body weight, and inflammation [76].

## 5. Gut–Brain Axis

Appetite regulation involves complex interactions of homeostatic and hedonic factors. Hypothalamus is central in the homeostatic control of food intake and other neural circuits integrate environmental and emotional cues to constitute the hedonic drive of appetite regulation. The ARC of the hypothalamus plays a pivotal role in the integration of signals regulating appetite. Due to the presence of a relatively leaky blood–brain barrier (BBB) and the ARC proximity to the median eminence (ME), circulating peripheral hormonal signals have relatively greater access to receptors in the ARC than other brain areas [38]. ARC comprises neurons that integrate signals of nutritional status and energy expenditure. Neurons that express POMC and CART are anorexigenic, whereas neurons that express (AgRP) and (NPY) are orexigenic [77]. Peripheral hormonal signals act on the hypothalamus to inhibit or activate ARC neurons to alter appetite. Further, ARC neurons also communicate with other orexigenic and anorexigenic neurons in other nuclei of the hypothalamus to regulate food intake [78]. Reward pathways activated in response to food comprise the dopaminergic neurons that originate in the VTA and *substantia nigra* (SN) in the midbrain with neuronal projections throughout the brain. Dopaminergic signals are received and integrated into the *nucleus accumbens*, striatum, and orbitofrontal cortex (OFC). These regions are activated in response to food cues and food intake during functional magnetic resonance imaging (fMRI) scans in humans [79]. Homeostatic and hedonic systems are integrated via the lateral hypothalamic area (LHA) [80] and through its projections regulate the VTA and brainstem nuclei such as the NTS, which is pivotal in the modulation of gut hormones and satiety signals [81]. CCK, GLP-1, and PYY also stimulate the reward circuitry either directly or through projections from the hypothalamus and brainstem nuclei such as the NTS [82]. A systematic review of (fMRI) studies has shown associations between ghrelin, leptin, CCK, GLP-1, and PYY with activations in the hypothalamus, OFC, ACC, insula, and amygdala. These findings reflect gut–brain interactions during food intake within the homeostatic regulatory framework [83]. Following food ingestion, the circulating adipose signals (ghrelin and insulin) cross the BBB and stimulate receptors on neurons in the hypothalamus. Satiety signals generated by ingested food penetrate subcortical areas, such as amygdala and striatum, regulating how much food is consumed. The hypothalamus then sends signals to cortical areas, such as the OFC, ACC, and insula, as part of the reward circuitry, where cognitive factors and adiposity signals are integrated. Finally, a higher order cognitive evaluation determines the individuals eating behavior [83].

Functional MRI studies have also reported that brain responses to a test meal are not just a function of calorie intake, but nutrient specific, with differential brain responses to sugars, lipids, and proteins being reported [84]. Most studies have demonstrated a glucose-induced decrease in hypothalamic neural response [85,86,87,88,89,90,91,92], with a few exceptions [93,94]. Glucose ingestion shows a stronger reduction in neural response than intravenous administration, indicating that the incretin effect is important in the hypothalamic response [88]. Glucose-mediated neural responses are CCK-independent, unlike lipid-mediated neural responses [89]. Unlike glucose, lipid-driven neural responses are abolished by the CCK1 receptor antagonist dexloxiglumide, indicating a CCK-dependent pathway [95]. Lipid-activated neural responses are also suppressed by ghrelin [96], indicating ghrelin’s role in attenuating the gut-derived satiety signals. In general, fats attenuate the neural response in reward areas of the brain [97,98], exerting stronger satiating effects, with a role in regulating mood possibly explaining comfort eating behaviors [97].

The traditional framework forming the basis of ingestive behavior studies implicate that pleasure derived from eating overrides homeostatic circuits, whereby the palatable properties of caloric foods overrule physiological negative feedback that restricts overeating, resulting in positive energy balance and weight gain [99,100]. This assumption has recently been challenged by evidence that physiological signals act independently of pleasure sensations derived from eating and are the major driving force in the ingestive decision-making process [101]. Subcortical gut–brain pathways sense nutritive properties independent of palatable properties of food and activate brain reward circuits, as demonstrated by studies using the flavor-nutrient conditioning paradigm, where flavors are paired with nutrients or non-nutrient solutions and administered to humans [102,103]. In the flavor-nutrient conditioning experiments, the striatal and hypothalamic responses are associated with nutrient sensing and metabolism when flavors are consumed with calories during conditioning [104]. These studies highlight the significance of gut hormones in activating the reward systems of the brain and that unconscious motivation for energy content is not related to the hedonic aspect of food [101]. Palatability may dictate what one eats rather than reflect how much one eats. IBD patient’s dietary choices may be governed by sensory properties of food as evidenced by their greater craving for sweet/savory foods relative to controls [11]. Palatable food choices in CD patients may be influenced by low mood, impaired inhibition control, and altered gut–brain signaling.

## 6. Intestinal Inflammation and Modulation of EEC Peptides

### 6.1. Inflammatory Response and Body Weight

The inflammatory response *per se* may be a cause of weight loss in IBD. Hypophagic eating behavior during intestinal inflammation may be partly attributed to the increase in proinflammatory cytokines such as IL-1 [105], IL-6 [106], and TNFα [107]. These regulators of the inflammatory response may influence metabolism, induce fever, and result in loss of appetite [108]. Intracerebroventricular infusion of pro-inflammatory cytokines such as TNFα and IL-1β reduce food intake in rats, indicating that inflammation may play a role in the central regulation of appetite [109]. In gastrointestinal disorders, cytokine-induced alteration of gastrointestinal motility inhibits feeding. For example, IL-1, TNFα decreases, and IL-10 increases gastrointestinal motility [110,111]. The modulation of intestinal motility by cytokines, maybe due to direct action in the GI system or an action mediated by the brain with efferent signaling via the autonomic nervous system [111].

### 6.2. Examples of Intestinal Inflammation in Mice and Humans

The role of the EEC axis in the induction of altered eating behavior has been described in a *Trichinella spiralis*-induced intestinal inflammation murine model [112]. In this study, mice were infected with T spiralis by oral gavage of 300 larvae, followed by measurement of food intake over 30 days. The peak of severity of intestinal inflammation was observed 9 days post-infection, which coincided with reduced food intake, weight loss, and CCK upregulation. This finding may be explained by the juxtaposition of the intestinal inflammation and the anatomical location of the CCK-producing I cells. Reduced food intake was further observed 25 days post-infection in the absence of CCK upregulation, due to an extraintestinal inflammatory response induced by T spiralis larvae encyst in the skeleton muscle. Inhibition of CCK receptors by loxiglumide improved food intake 9 days post-infection. However, inhibition of CCK receptors during the extraintestinal inflammatory phase showed no effect on feeding. Taken together, these findings indicate that CCK directly contributes to intestinal inflammation-induced hypophagia, but does not mediate hypophagia induced by extraintestinal inflammation [112].

Early studies of acute intestinal infection demonstrated that patients suffering from tropical malabsorption had elevated levels of gut hormones [113]. A significant increase in postprandial CCK was observed in patients with giardiasis [45]. After treatment with tinidazole, plasma CCK levels were normalized and symptoms such as nausea, bloating, and anorexia in these patients were ameliorated [45]. It has been suggested that the endogenous increase in CCK may contribute to symptoms such as nausea and discomfort observed during intestinal inflammation [45]. Moreover, CCK was also found to reduce bacterial translocation [114] and increase luminal IgA secretion [115]. Therefore, a reduction in food intake during a gut infection may be an immunological response that acts as a protective mechanism against environmental contaminants.

### 6.3. EEC Peptides and CD

Upregulation of EEC and EEC peptides has been reported in ileal CD. A study [116] investigating EEC subtypes and EEC markers using terminal ileal tissue from patients with (i) active ileal CD (*n* = 38), (ii) inactive ileal CD (*n* = 5), and colonic tissue from patients with (iii) active colonic CD (*n* = 12) (iv) inactive colonic CD (*n* = 4) and controls (*n* = 60) reported a 3.3-fold increase in CgA mRNA expression, 3.1 fold increase in GLP-1 expression, a 1.8 fold increase in neurogenin-3 (Ngn-3) gene expression (a transcription factor involved in the epithelial cell differentiation to the EEC lineage) in ileal CD compared with controls. Further, paired like homeobox 2b (Phox2b) (another gene implicated in CD) was found to be co-localized to EEC and showed a 1.5-fold increase in ileal CD. A significant increase in terminal ileal chromagranin A (CgA) expressing cells, in active ileal CD tissues was observed compared with controls. No change in CgA expressing cells was found in the active colonic CD tissues. A 2.5-fold increase in GLP-1-positive cells was seen in the active ileal CD tissues compared with control tissues. However, no difference in terminal ileal PYY expressing cells or mRNA expression was seen between active ileal CD tissue and controls. These findings collectively indicate an upregulation of EEC in ileal CD.

Investigation of EEC peptides and appetite-related symptoms in response to a mixed nutrient test meal in active ileal CD patients (*n* = 12), inactive ileal CD patients (*n* = 6), active colonic CD patients (*n* = 5), and controls (*n* = 13) showed a 2-fold increase in pre- and postprandial total PYY plasma levels in active ileal CD patients compared with controls and active colonic CD patients [117]. Plasma PYY positively correlated with symptoms of nausea and bloating in the ileal CD patients. No difference in pre- or postprandial active GLP-1 levels was seen between the groups. It was postulated that GLP-1 was less likely to play a part in altered appetite in CD. Further, a paradoxical non-significant postprandial elevation in ghrelin was found in the active ileal CD group. The reassessment of active ileal CD patients in remission showed that the postprandial PYY and ghrelin levels reverted to normal control levels, however, this finding is limited by the small number of patients restudied in remission (*n* = 6).

A further study [118] investigating postprandial gut hormone levels and gastric emptying as assessed by 13C-octanoic acid breath test, in response to a standardized breakfast meal in moderately active CD patients (*n* = 13) (4 ileal, 4 colonic, and 5 ileal-colonic), in active UC patients (*n* = 10), and diverticulitis patients (*n* = 7) found a 3-fold increase in postprandial plasma CCK levels compared with controls and was associated with delayed gastric emptying. Postprandial CCK plasma concentrations were significantly higher in CD patients with exclusively ileal CD compared with the patients with colonic and ileal-colonic CD, but this finding was again limited by the number of exclusively ileal CD patients (*n* = 4). No difference in postprandial PYY and GLP-1 was found between CD and controls.

Another study [119] also investigating postprandial gastric emptying as measured by 13C-octanoic acid breath test and gut hormones response to a test meal in controls (*n* = 24), active CD patients (*n* = 14), and active UC patients (*n* = 14) found that fasting CCK levels and maximal postprandial concentrations were similar between IBD patients and controls, however, patients with UC had significantly lower postprandial CCK levels than controls and CD patients. No association between CCK plasma levels and gastric emptying was found contradicting the previous finding by this group [118]. The authors attributed this inconsistency to the test meal composition containing less lipids and protein, that primarily stimulate CCK, also this study only had one patient with ileal CD, which may explain the absence of difference between groups. Fasting GLP-1, PYY, and postprandial PYY were similar between IBD and controls. However, postprandial GLP-1 responses were increased in IBD (including CD-control and UC-control) and were associated with delayed gastric emptying. The reassessment of IBD patients in remission showed accelerated gastric emptying and normal postprandial increase in GLP-1, indicating that the increased release of GLP-1 from the inflamed gut mucosa in IBD may result in delayed gastric emptying.

A decrease in fasting small bowel motility as measured by MRI has been observed in ileal CD patients, attributed to increased fasting GLP-1 and PYY levels relative to controls [120]. Further, CD patients also showed elevated fasting and postprandial adverse GI symptoms such as fullness, distention, and abdominal pain compared with controls [120]. The adverse symptoms observed in the postprandial phase may be EEC peptide-mediated alterations in the gut–brain axis, as opposed to altered intestinal physiology.

Taken together, these findings suggest elevated EEC activity in ileal CD. Elevation in EEC expression at the tissue and plasma level in ileal CD might affect appetite regulation through changes in CNS signaling pathways. Notably, the increase in tissue and plasma EEC peptide expression was only observed in patients with ileal CD, whereas patients with exclusive colonic CD were found to have a normal ileal expression of EEC peptides and normal postprandial responses to GLP-1 and PYY [116,117], suggesting that disease location may differentially affect postprandial EEC peptide responses to a meal.

### 6.4. Modulation of Gut–Brain Signaling in CD

Despite the difference in pathophysiology, similar EEC peptide elevations in PYY, GLP-1, CCK, and decrease in ghrelin have been found in patients with obesity that have undergone Roux-en-Y gastric bypass (RYGB) surgery (which involves bypassing most of the stomach and first section of the small intestine) [121,122]. Postoperatively, these obese patients exhibit lower activation in brain-hedonic responses to food in relation to elevated postprandial gut hormones [123,124,125]. Further, an increase in plasma PYY and GLP-1 levels were found following an ad libitum test meal in obese patients that underwent RYGB relative to patients with gastric banding surgery and BMI matched controls [126]. The somatostatin analogue, octreotide-induced suppression of postprandial PYY and GLP-1 after RYGB correlated positively with an increase in neural signal in the brain reward system, indicating the role of GLP-1 and PYY in the hedonic response to food [126]. Similarly, after RYGB surgery, inhibition of GLP-1 using receptor antagonist exendin 9–39, resulted in increased food cue-induced neural response in the caudate nucleus and increased activation in the insula in response to chocolate milk. The caudate and insula had shown a decrease in neural response, in the absence of the GLP-1 receptor antagonist, indicating the role of GLP-1 in reward-driven food intake [124]. Similar findings have been reported following the parenteral administration of GLP-1 and PYY in healthy subjects [127]. Fatty-acids, specifically those with an acyl chain C12 or more (e.g., dodecanoate) lead to a CCK-dependent increase in neural responses in the brainstem, the pons, hypothalamus, cerebellum, and the motor cortical areas [96]. Mechanistic inhibition through the CCK type 1 receptor inhibitor dexloxiglumide causes a reversal in neural responses and a normalization of appetite-related scores [96]. Although an exaggerated EEC peptide response to food intake has been described in ileal CD, the gut–brain axis has not yet been investigated. Future studies are warranted to investigate whether ileal CD patients have altered gut and brain responses to specific nutrients, due to upregulation in EEC peptides, resulting in changes in appetite and eating behavior.

## 7. The Effect of Intestinal and Systemic Inflammation on the CNS

Intestinal inflammation and abdominal pain may activate central sensitization pathways that convey visceral nociceptive afferent signals from the gut to the brain [128,129]. Additionally, psychosocial distress may also affect symptom perception and gut function [129]. These observations may imply a role for the CNS in chronic symptom generation and perception in IBD. Multiple factors such as systemic inflammatory response [130,131], fatigue [132,133], abdominal pain [134], psychological comorbidities [135], and medication use [136,137,138], may be linked with brain structural and functional abnormalities in IBD. Alterations in brain structure have been reported in CD patients with extraintestinal inflammation (EIM) relative to CD patients without EIM in the insula (pain processing) and the right ACC (emotion and impulse control), which may be attributed to systemic inflammation [131]. Multiple structural MRI, resting state, and task fMRI studies have been conducted in IBD. A meta-analysis investigating structural and functional brain changes in CD in remission found that CD patients had reduced resting-state functional connectivity in the paracentral lobule (motor function) and cingulate gyrus and reduced grey matter volume in the medial frontal gyrus (executive function) [139]. However, currently, there is a lack of food and eating behavior-specific studies, to understand the structural and functional brain changes associated with food intake and eating behavior in intestinal inflammation.

**Figure 1:**
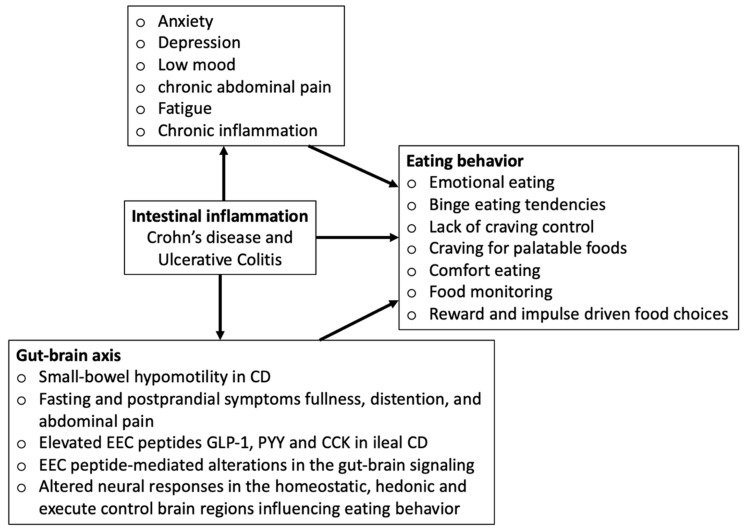
Conceptual framework.

The conceptual framework in Figure 1 depicts how intestinal inflammation-induced alterations in the gut–brain axis and disease symptoms may influence eating behaviors.

Table 1 provides a summary of the key studies investigating the upregulation of EEC peptides, altered eating behavior, and structural and functional brain changes in intestinal inflammation.

## 8. Conclusions

In summary, a complex interplay of gut peptides, altered gut–brain signaling, cognitive, psychological factors, disease-related symptoms, and inflammatory burden may ultimately guide eating behavior in intestinal inflammation. The studies mentioned in this review have several limitations. The studies investigating upregulation of EEC peptides in ileal CD are limited by sample size, patient standardization with variations in distribution and severity of inflammation, concomitant medication use, genotypes and triggers, and the nutrient composition of test meals used to assess postprandial physiological effects. Presently, there is a lack of food and eating behavior-specific fMRI task studies in IBD. Hence, future research could investigate food-induced alterations in gut–brain signaling in IBD patients using fMRI, to better understand the role of the brain in appetite control in intestinal inflammation. Further, the use of food-based impulsivity tasks will aid the understanding of impulse-driven eating behaviors in IBD patients. More studies need to assess patients in both the active phase of the disease and in remission, to see if the behavioral and CNS changes are reversible and associated with improved disease outcomes. Future IBD studies could use a non-gastrointestinal chronic disease group (such as rheumatoid arthritis or psoriasis) as comparator groups to dissect the relationship between gastrointestinal, systemic inflammation, and structural/functional brain alterations. Longitudinal studies are warranted to understand the effect of diet and disordered eating behaviors on disease course. A better understanding of the role of EEC peptides in altered eating behavior, malnutrition, and in the modulation of the gut–brain axis is relevant in elucidating new therapeutic pathways in CD treatment, thus improving nutritional status, disease outcomes, and quality of life. Dietetic advice for healthy eating is recommended to improve disordered eating traits. Psychological, cognitive, and behavioral therapeutic interventions may be beneficial to manage disease-related symptoms such as low mood, chronic fatigue, abdominal pain, increased self-monitoring, and impulsivity that may negatively influence eating behaviors.

## Figures and Tables

**Table 1:** Summary of key studies investigating the upregulation of EEC peptides, altered eating behavior, and structural and functional brain changes in intestinal inflammation.

Author, Country	Study Sample	Methods	Main Findings
Keller et al. 2009, GERMANY [118]	Active CD patients (*n* = 13) (4 ileal, 4 colonic, and 5 ileal-colonic), in active UC patients (*n* = 10), diverticulitis patients (*n* = 7)and HCs (*n* = 13)	Postprandial gut hormone levels (assessed by ELISA) and gastric emptying (assessed by 13C-octanoic acid breath test), in response to a standardized breakfast meal	A 3-fold increase in postprandial plasma CCK levels was found in active CD patients compared with HCs and was associated with delayed gastric emptying. Postprandial CCK plasma concentrations were significantly higher in CD patients with exclusively ileal CD compared with the patients with colonic and ileal-colonic CD. No difference in postprandial PYY and GLP-1 was found between CD and HCs.
Moran et al. 2012, UK [116]	Terminal ileal tissue from patients with (i) active ileal CD (*n* = 38), (ii) inactive ileal CD (*n* = 5), and colonic tissue from patients with (iii) active colonic CD (*n* = 12) (iv) inactive colonic CD (*n* = 4) and (v) HCs (*n* = 60)	Terminal ileal tissue from small or large bowel CD and HCs was analyzed for enteroendocrine marker expression by immunohistochemistry and quantitative polymerase chain reaction. Inflammation was graded by endoscopic, clinical, histological, and biochemical scoring	In ileal CD, GLP-1 and chromogranin A cells were increased 2.5-fold (*p* = 0.049) and 2-fold (*p* = 0.031), respectively. PYY cells were unchanged. Ileal EEC expression was unaffected in the presence of colonic CD. Phox2b was co-localized to EEC and showed a 1.5-fold increase in ileal disease. Significant mRNA increases were noted for chromogranin A (3.3-fold; *p* = 0.009), glucagon-like peptide 1 (3.1-fold; *p* = 0.007), and ubiquitination protein 4a (2.2-fold; *p* = 0.02). Neurogenin 3, an enteroendocrine transcription factor showed a 2-fold upregulation (*p* = 0.048).
Moran et al. 2013, UK [117]	Active ileal CD patients (*n* = 12), inactive ileal CD patients (*n* = 6), active colonic CD patients (*n* = 5), and HCs (*n* = 13)	Gut peptide responses to a mixed nutrient test meal were measured by ELISA. Symptoms were assessed by visual analogue score. A patient subset was re-studied in remission.	Ileal and colonic CD subjects displayed reduced appetite (*p* < 0.0001) before and after eating a mixed nutrient test meal compared with HCs. Total PYY was increased 2.2-fold (*p* = 0.04) and correlated with nausea (*p* = 0.036) and bloating (*p* = 0.037) scores only in small bowel CD compared with HCs. GLP-1 and GIP were not elevated. In remission, postprandial PYY and ghrelin reverted to control levels.
Keller et al. 2015, GERMANY [119]	Active CD patients (*n* = 14), active UC patients (*n* = 14), and HCs (*n* = 24)	Postprandial gastric emptying (measured by 13C-octanoic acid breath test), and gut hormone levels (measured by ELISA) in response to a test meal	Fasting CCK levels and maximal postprandial concentrations were similar between IBD patients and controls, however, patients with UC had significantly lower postprandial CCK levels than the controls and CD patients. No association between CCK plasma levels and gastric emptying was found. Fasting GLP-1, PYY, and postprandial PYY were similar between IBD and HCs. Postprandial GLP-1 responses were increased in IBD (including CD-control and UC-control) and were associated with delayed gastric emptying. The reassessment of IBD patients in remission showed accelerated gastric emptying and normal postprandial increase in GLP-1, indicating that the increased release of GLP-1 from the inflamed gut mucosa in IBD may result in delayed gastric emptying.
Wardle et al. 2018, UK [11]	Active CD patients (*n* = 30); HC (*n* = 31)	Disordered eating was assessed using validated questionnaires: Binge Eating Scale (BES); Power of Food Scale (PFS); Control of Eating Questionnaire (CoEQ); Dutch Eating Behavior Questionnaire (DEBQ); and Three Factor Eating Questionnaire (TFEQ). Food intake was assessed by 24-h dietary recall	Protein intake was lower in the CD cohort (*p* = 0.03) compared with HCs. Hospital Anxiety and Depression score was higher (*p* = 0.01) and CoEQ-Positive Mood (*p* = 0.001) lower in CD compared with HCs. CD patients were characterized by higher BES (*p* = 0.01) and lower CoEQ Craving Control (*p* = 0.027), with greater craving for Sweet (*p* = 0.043) and Savory (*p* = 0.021) foods relative to HCs. PFS food Present (food available but not physically present) (*p* = 0.005), DEBQ Emotional (*p* =< 0.001), and External Eating (the eating behavior triggered by external environmental stimuli, particularly, the presence of food, smell and/or taste, or even the time of day) (*p* = 0.022) were significantly higher than among HCs.
Khalaf et al. 2020, UK [120]	Active CD patients (*n* = 15) and 20 HCs (*n* = 20)	Small bowel motility (measured by MRI) and gut hormone levels (measured by ELISA) in response to a test meal	A decrease in fasting small bowel motility was observed in ileal CD patients compared with HCs. Fasting concentrations of GLP-1 and PYY were significantly greater in CD participants, compared with HCs (*p* ≤ 0.0001). The meal challenge induced a significant postprandial increase in aversive symptom scores (fullness, distention, bloating, abdominal pain, and sickness) in CD participants compared with HCs (*p* ≤ 0.05)
Yeung et al. 2020, Hong Kong [139]	Sixteen original studies comprised a total of 865 participants, where CD patients in remission (*n* = 486) and HCs (*n* = 379) were meta-analyzed	Original studies published until 2019 were identified from Scopus, Web of Science, and PubMed databases and included into the analysis if they reported relevant results from task-related or resting state functional magnetic resonance imaging (fMRI or rsfMRI) or voxel-based morphometry (VBM), in the form of standardized brain coordinates based on whole-brain analysis. The brain coordinates and sample size of significant results were extracted from eligible studies to be meta-analyzed with the activation likelihood estimation method using the GingerALE software	Compared to HCs, patients with CD had reduced resting state brain connectivity in the paracentral lobule (motor function) and cingulate gyrus (emotion and impulse control) as well as reduced grey matter volume in the medial frontal gyrus (executive function).

EEC = enteroendocrine cells, HC = healthy controls, CD = Crohn’s disease, UC = ulcerative colitis, ELISA = enzyme linked immunosorbent assay, CCK = cholecystokinin, GLP-1 = Glucagon-like peptide, PYY = Peptide YY.

## Data Availability

N/A.

## References

[1] Poulia K.-A., Klek S., Doundoulakis I., Bouras E., Karayiannis D., Baschali A., Passakiotou M., Chourdakis M. (2017). The two most popular malnutrition screening tools in the light of the new ESPEN consensus definition of the diagnostic criteria for malnutrition. Clin. Nutr..

[2] Sonnenberg A., Collins J.F. (2006). Vicious circles in inflammatory bowel disease. Inflamm. Bowel Dis..

[3] Wędrychowicz A., Zając A., Tomasik P. (2016). Advances in nutritional therapy in inflammatory bowel diseases: Review. World J. Gastroenterol..

[4] De Graaf C., Blom W.A.M., Smeets P.A.M., Stafleu A., Hendriks H.F.J. (2004). Biomarkers of satiation and satiety. Am. J. Clin. Nutr..

[5] Bruen C.M., O’Halloran F., Cashman K.D., Giblin L. (2012). The effects of food components on hormonal signalling in gastrointestinal enteroendocrine cells. Food Funct..

[6] Satherley R.-M., Howard R., Higgs S. (2016). The prevalence and predictors of disordered eating in women with coeliac disease. Appetite.

[7] Satherley R., Howard R., Higgs S. (2015). Disordered eating practices in gastrointestinal disorders. Appetite.

[8] Limdi J.K., Aggarwal D., McLaughlin J.T. (2016). Dietary Practices and Beliefs in Patients with Inflammatory Bowel Disease. Inflamm. Bowel Dis..

[9] Casanova M.J., Chaparro M., Molina B., Merino O., Batanero R., Dueñas-Sadornil C., Robledo P., Garcia-Albert A.M., Gómez-Sánchez M.B., Calvet X. (2017). Prevalence of Malnutrition and Nutritional Characteristics of Patients with Inflammatory Bowel Disease. J. Crohn’s Colitis.

[10] De Vries J.H., Dijkhuizen M., Tap P., Witteman B.J. (2019). Patient’s Dietary Beliefs and Behaviours in Inflammatory Bowel Disease. Dig. Dis..

[11] Wardle R.A., Thapaliya G., Nowak A., Radford S., Dalton M., Finlayson G., Moran G.W. (2018). An Examination of Appetite and Disordered Eating in Active Crohn’s Disease. J. Crohn’s Colitis.

[12] Filippi J., Al-Jaouni R., Wiroth J.B., Hébuterne X., Schneider S.M. (2006). Nutritional deficiencies in patients with Crohn’s disease in remission. Inflamm. Bowel Dis..

[13] Aghdassi E., Wendland B.E., Stapleton M., Raman M., Allard J.P. (2007). Adequacy of Nutritional Intake in a Canadian Population of Patients with Crohn’s Disease. J. Am. Diet. Assoc..

[14] Wardle R.A., Wardle A.J., Charadava C., Ghosh S., Moran G.W. (2017). Literature review: Impacts of socioeconomic status on the risk of inflammatory bowel disease and its outcomes. Eur. J. Gastroenterol. Hepatol..

[15] Hindryckx P., Laukens D., D’Amico F., Danese S. (2018). Unmet Needs in IBD: The Case of Fatigue. Clin. Rev. Allergy Immunol..

[16] Pizzi L.T., Weston C.M., Goldfarb N.I., Moretti D., Cobb N., Howell J.B., Infantolino A., Dimarino A.J., Cohen S. (2006). Impact of chronic conditions on quality of life in patients with inflammatory bowel disease. Inflamm. Bowel Dis..

[17] Vavricka S.R., Schoepfer A., Scharl M., Lakatos P.L., Navarini A.A., Rogler G. (2015). Extraintestinal Manifestations of Inflammatory Bowel Disease. Inflamm. Bowel Dis..

[18] Van Oudenhove L., McKie S., Lassman D., Uddin B., Paine P., Coen S., Gregory L., Tack J., Aziz Q. (2011). Fatty acid–induced gut-brain signaling attenuates neural and behavioral effects of sad emotion in humans. J. Clin. Investig..

[19] Cavicchia P.P., Steck S.E., Hurley T.G., Hussey J.R., Ma Y., Ockene I.S., Hébert J.R. (2009). A new dietary inflammatory index predicts interval changes in serum high-sensitivity C-reactive protein. J. Nutr..

[20] Lamers C.R., De Roos N.M., Witteman BJ M. (2020). The association between inflammatory potential of diet and disease activity: Results from a cross-sectional study in patients with inflammatory bowel disease. BMC Gastroenterol..

[21] Dalley J.W., Everitt B.J., Robbins T.W. (2011). Impulsivity, Compulsivity, and Top-Down Cognitive Control. Neuron.

[22] Evenden J.L. (1999). Varieties of impulsivity. Psychopharmacology.

[23] Kagan J. (1966). Reflection-impulsivity: The generality and dynamics of conceptual tempo. J. Abnorm. Psychol..

[24] La Barbera D., Bonanno B., Rumeo M.V., Alabastro V., Frenda M., Massihnia E., Morgante M.C., Sideli L., Craxì A., Cappello M. (2017). Alexithymia and personality traits of patients with inflammatory bowel disease. Sci. Rep..

[25] Sybil B., GEysenck HJ E. (1978). Impulsiveness and venturesomeness: Their position in a dimensional system of personality. Psychol. Rep..

[26] Hyphantis T., Antoniou K., Tomenson B., Tsianos E., Mavreas V., Creed F. (2010). Is the personality characteristic ‘impulsive sensation seeking’ correlated to differences in current smoking between ulcerative colitis and Crohn’s disease patients?. Gen. Hosp. Psychiatry.

[27] Reuter M., Netter P. (2001). The influence of personality on nicotine craving: A hierarchical multivariate statistical prediction model. Neuropsychobiology.

[28] Berridge K.C., Robinson T.E., Aldridge J.W. (2010). Dissecting components of reward: ‘liking’, ‘wanting’, and learning. Curr. Opin. Pharmacol..

[29] Sternini C., Anselmia L., Rozengurt E. (2008). Enteroendocrine cells: A site of ‘taste’ in gastrointestinal chemosensing. Curr. Opin. Endocrinol. Diabetes Obes..

[30] Posovszky C., Wabitsch M. (2015). Regulation of appetite, satiation, and body weight by enteroendocrine cells. Part 1: Characteristics of enteroendocrine cells and their capability of weight regulation. Horm. Res. Paediatr..

[31] Date Y., Murakami N., Toshinai K., Matsukura S., Niijima A., Matsuo H., Kangawa K., Nakazato M. (2002). The role of the gastric afferent vagal nerve in ghrelin-induced feeding and growth hormone secretion in rats. Gastroenterology.

[32] Sukkar S.G., Vaccaro A., Ravera G.B., Borrini C., Gradaschi R., Sacchi-Nemours A.M., Cordera R., Andraghetti G. (2013). Appetite control and gastrointestinal hormonal behavior (CCK, GLP-1, PYY 1-36) following low doses of a whey protein-rich nutraceutic. Med. J. Nutr. Metab..

[33] Moran G.W., Leslie F.C., Levison S.E., Worthington J., McLaughlin J.T. (2008). Enteroendocrine cells: Neglected players in gastrointestinal disorders?. Therap. Adv. Gastroenterol..

[34] Furness J.B., Kunze W.A.A., Clerc N. (1999). The intestine as a sensory organ: Neural, endocrine, and immune responses. Am. J. Physiol. Liver Physiol..

[35] Berthoud H.-R., Neuhuber W.L. (2000). Functional and chemical anatomy of the afferent vagal system. Auton. Neurosci..

[36] Berthoud H.-R. (2008). Vagal and hormonal gut-brain communication: From satiation to satisfaction. Neurogastroenterol. Motil..

[37] Schwartz G.J. (2000). The role of gastrointestinal vagal afferents in the control of food intake: Current prospects. Nutrition.

[38] Perry B., Wang Y. (2012). Appetite regulation and weight control: The role of gut hormones. Nutr. Diabetes.

[39] Cummings D.E., Overduin J. (2007). Gastrointestinal regulation of food intake. J. Clin. Investig..

[40] McLaughlin J.T., Lomax R.B., Hall L., Dockray G.J., Thompson D.G., Warhurst G. (1998). Fatty acids stimulate cholecystokinin secretion via an acyl chain length-specific, Ca^2+^-dependent mechanism in the enteroendocrine cell line STC-1. J. Physiol..

[41] Liou A.P., Lu X., Sei Y., Zhao X., Pechhold S., Carrero R.J., Raybould H.E., Wank S. (2011). The G-protein-coupled receptor GPR40 directly mediates Long-chain fatty acid-induced secretion of cholecystokinin. Gastroenterology.

[42] Valassi E., Scacchi M., Cavagnini F. (2008). Neuroendocrine control of food intake. Nutr. Metab. Cardiovasc. Dis..

[43] Huda MS B., Wilding JP H., Pinkney J.H. (2006). Gut peptides and the regulation of appetite. Obes. Rev..

[44] Moran T., Sheng B. (2007). Hyperphagia and Obesity of OLETF Rats Lacking CCK1 Receptors: Developmental Aspects. Dev. Psychobiol..

[45] Leslie F.C., Thompson D.G., McLaughlin J.T., Varro A., Dockray G.J., Mandal B.K. (2003). Plasma cholecystokinin concentrations are elevated in acute upper gastrointestinal infections. QJM Mon. J. Assoc. Physicians.

[46] Crawley J.N., Corwin R.L. (1994). Biological actions of cholecystokinin. Peptides.

[47] Matson C.A., Reid D.F., Cannon T.A., Ritter R.C. (2000). Cholecystokinin and leptin act synergistically to reduce body weight. Am. J. Physiol. Regul. Integr. Comp. Physiol..

[48] Herrmann C., Göke R., Richter G., Fehmann H.-C., Arnold R., Göke B. (1995). Glucagon-Like Peptide-1 and Glucose-Dependent Insulin-Releasing Polypeptide Plasma Levels in Response to Nutrients. Digestion.

[49] Holmes G.M., Browning K.N., Tong M., Qualls-Creekmore E., Travagli R.A. (2009). Vagally mediated effects of glucagon-like peptide 1: In vitro and in vivo gastric actions. J. Physiol..

[50] Lim G.E., Brubaker P.L. (2006). Glucagon-like peptide 1 secretion by the L-cell: The view from within. Diabetes.

[51] Chaudhri O.B., Wynne K., Bloom S.R. (2008). Can gut hormones control appetite and prevent obesity?. Diabetes Care.

[52] Sisley S., Gutierrez-Aguilar R., Scott M., D’Alessio D.A., Sandoval D.A., Seeley R.J. (2014). Neuronal GLP1R mediates liraglutide’s anorectic but not glucose-lowering effect. J. Clin. Investig..

[53] Baggio L.L., Drucker D.J. (2014). Glucagon-like peptide-1 receptors in the brain: Controlling food intake and body weight. J. Clin. Investig..

[54] Secher A., Jelsing J., Baquero A.F., Hecksher-Sørensen J., Cowley M.A., Dalbøge L.S., Hansen G., Grove K.L., Pyke C., Raun K. (2014). The arcuate nucleus mediates GLP-1 receptor agonist liraglutide-dependent weight loss. J. Clin. Investig..

[55] Farr O.M., Sofopoulos M., Tsoukas M.A., Dincer F., Thakkar B., Sahin-Efe A., Filippaios A., Bowers J., Srnka A., Gavrieli A. (2016). GLP-1 receptors exist in the parietal cortex, hypothalamus and medulla of human brains and the GLP-1 analogue liraglutide alters brain activity related to highly desirable food cues in individuals with diabetes: A crossover, randomised, placebo-controlled trial. Diabetologia.

[56] Kulve J.S.T., Veltman D.J., Van Bloemendaal L., Groot P.F.C., Ruhé H.G., Barkhof F., Diamant M., Ijzerman R.G. (2016). Endogenous GLP1 and GLP1 analogue alter CNS responses to palatable food consumption. J. Endocrinol..

[57] Filippatos T.D., Panagiotopoulou T.V., Elisaf M.S. (2015). Adverse Effects of GLP-1 Receptor Agonists. Rev. Diabet. Stud..

[58] Batterham R.L., Cowley M.A., Small C.J., Herzog H., Cohen M.A., Dakin C.L., Wren A.M., Brynes A.E., Low M.J., Ghatei M.A. (2004). Does gut hormone PYY3-36 decrease food intake in rodents? (reply). Nature.

[59] Anini Y., Fu-Cheng X., Cuber J.C., Kervran A., Chariot J. (1999). Comparison of the postprandial release of peptide YY and proglucagon- derived peptides in the rat. Pflug. Arch. Eur. J. Physiol..

[60] Lin H.C., Chey W.Y., Zhao X. (2000). Release of distal gut peptide YY (PYY) by fat in proximal gut depends on CCK. Peptides.

[61] Kim B.-J., Carlson O.D., Jang H.-J., Elahi D., Berry C., Egan J.M. (2005). Peptide YY is secreted after oral glucose administration in a gender-specific manner. J. Clin. Endocrinol. Metab..

[62] Lumb K.J., DeCarr L.B., Milardo L.F., Mays M.R., Buckholz T.M., Fisk S.E., Pellegrino C.M., Ortiz A.A., Mahle C.D. (2007). Novel selective neuropeptide Y2 receptor PEGylated peptide agonists reduce food intake and body weight in mice. J. Med. Chem..

[63] Abbott C.R., Small C.J., Kennedy A.R., Neary N.M., Sajedi A., Ghatei M.A., Bloom S.R. (2005). Blockade of the neuropeptide Y Y2 receptor with the specific antagonist BIIE0246 attenuates the effect of endogenous and exogenous peptide YY (3-36) on food intake. Brain Res..

[64] Vincent R.P., le Roux C.W. (2008). The satiety hormone peptide YY as a regulator of appetite. J. Clin. Pathol..

[65] Koda S., Date Y., Murakami N., Shimbara T., Hanada T., Toshinai K., Niijima A., Furuya M., Inomata N., Osuye K. (2005). The role of the vagal nerve in peripheral PYY 3-36-induced feeding reduction in rats. Endocrinology.

[66] Savage A.P., Adrian T.E., Carolan G., Chatterjee V.K., Bloom S.R. (1987). Effects of peptide YY (PYY) on mouth to caecum intestinal transit time and on the rate of gastric emptying in healthy volunteers. Gut.

[67] le Roux C.W., Borg C.M., Murphy K.G., Vincent R.P., Ghatei M.A., Bloom S.R. (2008). Supraphysiological doses of intravenous PYY3-36 cause nausea, but no additional reduction in food intake. Ann. Clin. Biochem..

[68] Tschop M., Smiley D.L., Heiman M.L. (2000). Ghrelin induces adiposity in rodents. Nature.

[69] Chen H.Y., Trumbauer M.E., Chen A.S., Weingarth D.T., Adams J.R., Frazier E.G., Shen Z., Marsh D.J., Feighner S.D., Guan X.-M. (2004). Orexigenic action of peripheral ghrelin is mediated by neuropeptide Y and agouti-related protein. Endocrinology.

[70] Perez-Tilve D., Heppner K., Kirchner H., Lockie S.H., Woods S.C., Smiley D.L., Tschöp M., Pfluger P. (2011). Ghrelin-induced adiposity is independent of orexigenic effects. FASEB J..

[71] Yamazaki M., Nakamura K., Kobayashi H., Matsubara M., Hayashi Y., Kangawa K., Sakai T. (2002). Regulational effect of ghrelin on growth hormone secretion from perifused rat anterior pituitary cells. J. Neuroendocrinol..

[72] Chuang J.-C., Sakata I., Kohno D., Perello M., Osborne-Lawrence S., Repa J.J., Zigman J.M. (2011). Ghrelin directly stimulates glucagon secretion from pancreatic α-cells. Mol. Endocrinol..

[73] Tong J., Prigeon R.L., Davis H.W., Bidlingmaier M., Kahn S.E., Cummings D.E., Tschop M.H., D’Alessio D. (2010). Ghrelin suppresses glucose-stimulated insulin secretion and deteriorates glucose tolerance in healthy humans. Diabetes.

[74] Alfonso A. (2019). Stress and obesity: The ghrelin connection. J. Neuroendocrinol..

[75] Nikitopoulou I., Kampisiouli E., Jahaj E., Vassiliou A., Dimopoulou I., Mastora Z., Tsakiris S., Perreas K., Tzanela M., Routsi C. (2020). Ghrelin alterations during experimental and human sepsis. Cytokine.

[76] Baatar D., Patel K., Taub D.D. (2011). The effects of ghrelin on inflammation and the immune system. Mol. Cell. Endocrinol..

[77] Schwartz M.W., Porte D. (2005). Diabetes, Obesity, and the Brain. Science.

[78] Farr O.M., Li C.-S.R., Mantzoros C.S. (2016). Central nervous system regulation of eating: Insights from human brain imaging. Metabolism.

[79] DiLeone R.J., Taylor J.R., Picciotto M.R. (2012). The drive to eat: Comparisons and distinctions between mechanisms of food reward and drug addiction. Nat. Neurosci..

[80] Kelley A.E., Baldo B.A., Pratt W.E., Will M.J. (2005). Corticostriatal-hypothalamic circuitry and food motivation: Integration of energy, action and reward. Physiol. Behav..

[81] Morton G.J., Meek T.H., Schwartz M.W. (2014). Neurobiology of food intake in health and disease. Nat. Rev. Neurosci..

[82] Ziauddeen H., Alonso-alonso M., Hill J.O., Kelley M., Khan N.A. (2015). Obesity and the Neurocognitive Basis of Food Reward and the Control of Intake. Adv. Nutr..

[83] Zanchi D., Depoorter A., Egloff L., Haller S., Mählmann L., Lang U.E., Drewe J., Beglinger C., Schmidt A., Borgwardt S. (2017). The impact of gut hormones on the neural circuit of appetite and satiety: A systematic review. Neurosci. Biobehav. Rev..

[84] McLaughlin J.T., McKie S. (2016). Human brain responses to gastrointestinal nutrients and gut hormones. Curr. Opin. Pharmacol..

[85] Liu Y., Gao J.-H., Liu H.-L., Fox P.T. (2000). The temporal response of the brain after eating revealed by functional MRI. Nature.

[86] Smeets PA M., De Graaf C., Stafleu A., Van Osch MJ P., Van Der Grond J. (2005). Functional MRI of human hypothalamic responses following glucose ingestion. Neuroimage.

[87] Smeets P.A.M., de Graaf C., Stafleu A., van Osch M.J.P., van der Grond J. (2005). Functional magnetic resonance imaging of human hypothalamic responses to sweet taste and calories. Am. J. Clin. Nutr..

[88] Smeets P.A.M., Vidarsdottir S., De Graaf C., Stafleu A., Van Osch M.J.P., Viergever M.A., Pijl H., Van Der Grond J. (2007). Oral glucose intake inhibits hypothalamic neuronal activity more effectively than glucose infusion. Am. J. Physiol. Endocrinol. Metab..

[89] Little T.J., McKie S., Jones R.B., D’Amato M., Smith C.P., Kiss O., Thompson D.G., McLaughlin J.T. (2014). Mapping glucose-mediated gut-to-brain signalling pathways in humans. Neuroimage.

[90] Jastreboff A.M., Sinha R., Arora J., Giannini C., Kubat J., Malik S., Van Name M.A., Santoro N., Savoye M., Duran E.J. (2016). Altered brain response to drinking glucose and fructose in obese adolescents. Diabetes.

[91] Van Opstal A.M., van den Berg-Huysmans A.A., Hoeksma M., Blonk C., Pijl H., Rombouts S.A.R.B., van der Grond J. (2018). The effect of consumption temperature on the homeostatic and hedonic responses to glucose ingestion in the hypothalamus and the reward system. Am. J. Clin. Nutr..

[92] Van Opstal A., Kaal I., Berg-Huysmans A.V.D., Hoeksma M., Blonk C., Pijl H., Rombouts S., Van Der Grond J. (2019). Dietary sugars and non-caloric sweeteners elicit different homeostatic and hedonic responses in the brain. Nutrition.

[93] Purnell J.Q., Klopfenstein B.A., Stevens A.A., Havel P.J., Adams S.H., Dunn T.N., Krisky C., Rooney W.D. (2011). Brain functional magnetic resonance imaging response to glucose and fructose infusions in humans. Diabetes Obes. Metab..

[94] Stopyra M.A., Friederich H.-C., Sailer S., Pauen S., Bendszus M., Herzog W., Simon J.J. (2019). The effect of intestinal glucose load on neural regulation of food craving. Nutr. Neurosci..

[95] Lassman D.J., McKie S., Gregory L.J., Lal S., D’Amato M., Steele I., Varro A., Dockray G.J., Williams S.C., Thompson D.G. (2010). Defining the Role of Cholecystokinin in the Lipid-Induced Human Brain Activation Matrix. Gastroenterology.

[96] Jones R.B., McKie S., Astbury N., Little T.J., Tivey S., Lassman D.J., McLaughlin J., Luckman S., Williams S.R., Dockray G.J. (2012). Functional neuroimaging demonstrates that ghrelin inhibits the central nervous system response to ingested lipid. Gut.

[97] Eldeghaidy S., Marciani L., Hort J., Hollowood T., Singh G., Bush D., Foster T., Taylor A.J., Busch J., Spiller R.C. (2016). Prior Consumption of a Fat Meal in Healthy Adults Modulates the Brain’s Response to Fat. J. Nutr..

[98] Frank-Podlech S., Heinze J.M., Machann J., Scheffler K., Camps G., Fritsche A., Rosenberger M., Hinrichs J., Veit R., Preissl H. (2019). Functional Connectivity within the Gustatory Network Is Altered by Fat Content and Oral Fat—A Pilot Study. Front. Neurosci..

[99] Cifford S.B., Chou T.C., Elmquist J.K. (2002). The Need to Feed: Homeostatic and Hedonic Control of Eating. Neuron.

[100] Rossi M.A., Stuber G.D. (2018). Overlapping Brain Circuits for Homeostatic and Hedonic Feeding. Cell Metab..

[101] de Araujo I.E., Schatzker M., Small D.M. (2020). Rethinking Food Reward. Annu. Rev. Psychol..

[102] Yeomans M.R., Leitch M., Gould N.J., Mobini S. (2008). Differential hedonic, sensory and behavioral changes associated with flavor-nutrient and flavor-flavor learning. Physiol. Behav..

[103] Brunstrom J.M., Mitchell G.L. (2007). Flavor-nutrient learning in restrained and unrestrained eaters. Physiol. Behav..

[104] De Araujo I.E., Lin T., Veldhuizen M.G., Small D.M. (2013). Metabolic regulation of brain response to food cues. Curr. Biol..

[105] Mahida Y.R., Wu K., Jewell D.P. (1989). Enhanced production of interleukin 1-beta by mononuclear cells isolated from mucosa with active ulcerative colitis of Crohn’s disease. Gut.

[106] Mahida Y.R., Kurlac L., Gallagher A., Hawkey C.J. (1991). High circulating concentrations of interleukin-6 in active Crohn ’ s disease but not ulcerative colitis. Gut.

[107] Macdonald T.T., Hutchings P., Choy M., Murch S., Cooke A. (1990). Tumour necrosis factor-alpha and interferon-gamma production measured at the single cell level in normal and inflamed human intestine. Clin. Exp. Immunol..

[108] Chrousos G. (1995). The Hypothalmic-pituitary-adrenal axis and immune-mediated inflammation. N. Engl. J. Med..

[109] Plata-Salaman C.R., Oomura Y., Kai Y. (1988). Tumor necrosis factor and interleukin-1 beta: Suppression of food intake by direct action in the central nervous system. Brain Res.

[110] Akiho H. (2011). Cytokine-induced alterations of gastrointestinal motility in gastrointestinal disorders. World J. Gastrointest. Pathophysiol..

[111] Plata-Salamán C.R. (2001). Cytokines and feeding. Int. J. Obes..

[112] Mcdermott J.R., Leslie F.C., Thompson D.G., Grencis R.K., Mclaughlin J.T. (2006). Immune control of food intake: Enteroendocrine cells are regulated by CD4+ T lymphocytes during small intestinal inflammtion. Neurogastroenterology.

[113] Besterman H.S., Cook G.C., Sarson D.L., Christofides N.D., Bryant M.G., Gregor M., Bloom S.R. (1979). Gut hormones in tropical malabsorption. Br. Med. J..

[114] Solteszb X.W., Andersson J.A.R. (1996). Cholecystokinin increases small intestinal motility and reduces enteric bacterial overgrowth and translocation in rats with surgically induced acute liver failure. Digestion.

[115] Freier S., Eran M., Faber J. (1987). Effect of cholecystokinin and of its antagonist, of atropine, and of food on the release of immunoglobulin A and immunoglobulin G specific antibodies in the rat intestine. Gastroenterology.

[116] Moran G.W., Pennock J., Mclaughlin J.T. (2012). Enteroendocrine cells in terminal ileal Crohn’s disease. J. Crohn’s Colitis.

[117] Moran G.W., Leslie F.C., McLaughlin J.T. (2013). Crohn’s disease affecting the small bowel is associated with reduced appetite and elevated levels of circulating gut peptides. Clin. Nutr..

[118] Keller J., Beglinger C., Holst J.J., Andresen V., Layer P. (2009). Mechanisms of gastric emptying disturbances in chronic and acute inflammation of the distal gastrointestinal tract. Am. J. Physiol. Gastrointest. Liver Physiol..

[119] Keller J., Binnewies U., Rösch M., Holst J.J., Beglinger C., Andresen V., Layer P. (2015). Gastric emptying and disease activity in inflammatory bowel disease. Eur. J. Clin. Investig..

[120] Khalaf A., Hoad C.L., Menys A., Nowak A., Radford S., Taylor S.A., Latief K., Lingaya M., Falcone Y., Singh G. (2019). Gastrointestinal peptides and small-bowel hypomotility are possible causes for fasting and postprandial symptoms in active Crohn’s disease. Am. J. Clin. Nutr..

[121] Jacobsen S.H., Olesen S.C., Dirksen C., Jørgensen N.B., Bojsen-Møller K.N., Kielgast U., Worm D., Almdal T., Naver L.S., Hvolris L.E. (2012). Changes in gastrointestinal hormone responses, insulin sensitivity, and beta-cell function within 2 weeks after gastric bypass in non-diabetic subjects. Obes. Surg..

[122] Dirksen C., Jørgensen N.B., Bojsen-Møller K.N., Kielgast U., Jacobsen S.H., Clausen T.R., Worm D., Hartmann B., Rehfeld J.F., Damgaard M. (2013). Gut hormones, early dumping and resting energy expenditure in patients with good and poor weight loss response after Roux-en-Y gastric bypass. Int. J. Obes..

[123] Scholtz S., Miras A.D., Chhina N., Prechtl C.G., Sleeth M.L., Daud N.M., Ismail N.A., Durighel G., Ahmed A.R., Olbers T. (2014). Obese patients after gastric bypass surgery have lower brain-hedonic responses to food than after gastric banding. Gut.

[124] Kulve J.S.T., Veltman D.J., Gerdes V.E., Van Bloemendaal L., Barkhof F., Deacon C.F., Holst J.J., Drent M.L., Diamant M., Ijzerman R.G. (2017). Elevated postoperative endogenous GLP-1 levels mediate effects of roux-en-Y gastric bypass on neural responsivity to food cues. Diabetes Care.

[125] Zoon H.F., De Bruijn S.E., Smeets P.A., De Graaf C., Janssen I.M., Schijns W., Aarts E.O., Jager G., Boesveldt S. (2018). Altered neural responsivity to food cues in relation to food preferences, but not appetite-related hormone concentrations after RYGB-surgery. Behav. Brain Res..

[126] Goldstone A.P., Miras A.D., Scholtz S., Jackson S., Neff K.J., Pénicaud L., Geoghegan J., Chhina N., Durighel G., Bell J.D. (2016). Link between increased satiety gut hormones and reduced food reward following gastric bypass surgery for obesity. J. Clin. Endocrinol. Metab..

[127] De Silva A., Salem V., Long C.J., Makwana A., Newbould R.D., Rabiner E.A., Ghatei M.A., Bloom S.R., Matthews P.M., Beaver J.D. (2011). The Gut Hormones PYY3-36 and GLP-17-36 amide Reduce Food Intake and Modulate Brain Activity in Appetite Centers in Humans Akila. Cell Metab..

[128] Hubbard C.S., Becerra L., Heinz N., Ludwick A., Rasooly T., Wu R., Johnson A., Schechter N.L., Borsook D., Nurko S. (2016). Abdominal pain, the adolescent and altered brain structure and function. PLoS ONE.

[129] Jones M.P., Dilley J.B., Drossman D., Crowell M.D. (2006). Brain-gut connections in functional GI disorders: Anatomic and physiologic relationships. Neurogastroenterol. Motil..

[130] Dantzer R., O’Connor J.C., Freund G.G., Johnson R.W., Kelley K.W. (2008). From inflammation to sickness and depression: When the immune system subjugates the brain. Nat. Rev. Neurosci..

[131] Thomann A.K., Thomann P.A., Wolf R.C., Hirjak D., Schmahl C., Ebert M.P., Szabo K., Reindl W., Griebe M. (2016). Altered markers of brain development in Crohn’s disease with extraintestinal manifestations—A pilot study. PLoS ONE.

[132] Ratnakumaran R., Warren L., Gracie D.J., Sagar R.C., Hamlin P.J., Ford A.C., O’Connor A. (2018). Fatigue in Inflammatory Bowel Disease Reflects Mood and Symptom-Reporting Behavior Rather Than Biochemical Activity or Anemia. Clin. Gastroenterol. Hepatol..

[133] Van Erp S., Ercan E., Breedveld P., Brakenhoff L., Ghariq E., Schmid S., Van Osch M., Van Buchem M., Emmer B., Van Der Grond J. (2017). Cerebral magnetic resonance imaging in quiescent Crohn’s disease patients with fatigue. World J. Gastroenterol..

[134] Bao C., Liu P., Shi Y., Wu L., Jin X., Zeng X., Zhang J., Wang D., Liu H., Wu H. (2017). Differences in brain gray matter volume in patients with Crohn’s disease with and without abdominal pain. Oncotarget.

[135] Bao C.H., Liu P., Liu H.R., Wu L.Y., Shi Y., Chen W.F., Qin W., Lu Y., Zhang J.Y., Jin X.M. (2015). Alterations in brain gray matter structures in patients with Crohn’s disease and their correlation with psychological distress. J. Crohn’s Colitis.

[136] Goodhand J.R., Greig F.I., Koodun Y., McDermott A., Wahed M., Langmead L., Rampton D.S. (2012). Do antidepressants influence the disease course in inflammatory bowel disease? A retrospective case-matched observational study. Inflamm. Bowel Dis..

[137] Casella G., Tontini G.E., Bassotti G., Pastorelli L., Villanacci V., Spina L., Baldini V., Vecchi M. (2014). Neurological disorders and inflammatory bowel diseases. World J. Gastroenterol..

[138] Mrakotsky C., Anand R., Watson C., Vu C., Matos A., Friel S., Rivkin M., Snapper S. (2016). New Evidence for Structural Brain Differences in Pediatric Crohn’s Disease: Impact of Underlying Disease Factors. Inflamm. Bowel Dis..

[139] Yeung A.W.K. (2020). Structural and functional changes in the brain of patients with Crohn’s disease: An activation likelihood estimation meta-analysis. Brain Imaging Behav..

